# Supplementary material for: Reconstruction of Par-dependent polarity in apolar cells reveals a dynamic process of cortical polarization
Source: eLife. 2019 Jun 7;8:e45559. doi: 10.7554/eLife.45559 (PMC6555595; doi:10.7554/eLife.45559)
Supplement: Figure 3—source data 2. [file elife-45559-fig3-data2.pdf]

# UAS-Par3

(exp.1)

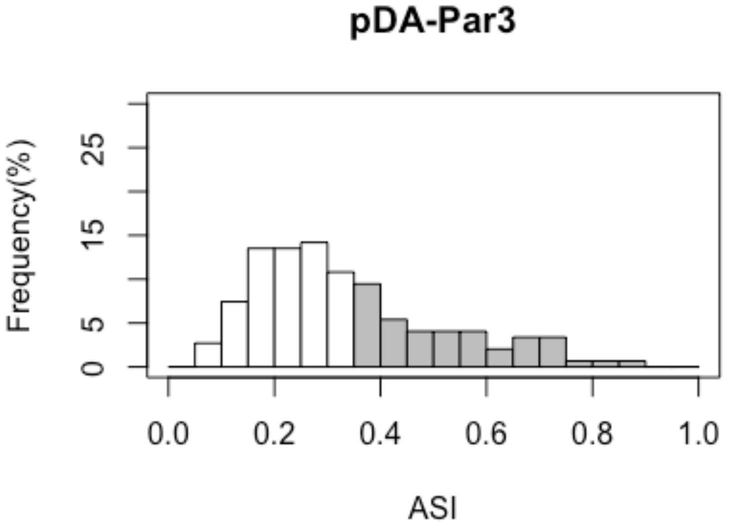


n=148
mean = 0.34, sd = 0.18
(ASI>0.35 mean = 0.52, sd = 0.14)

(exp.2)

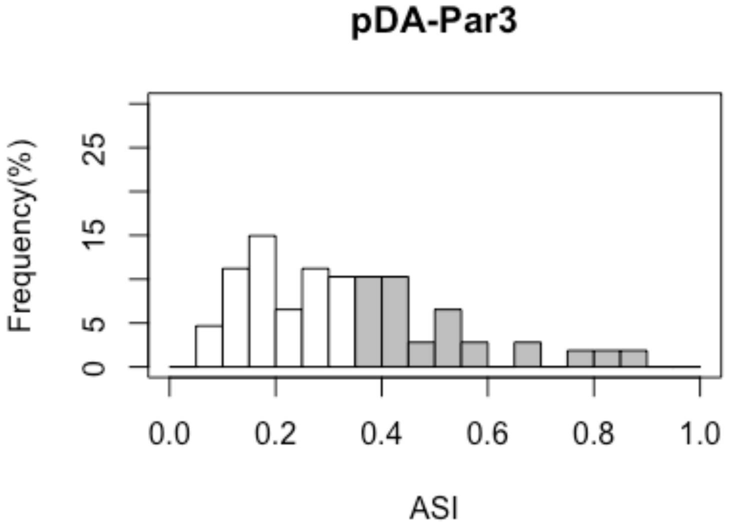


n=107
mean = 0.33, sd = 0.19
(ASI>0.35 mean = 0.52, sd = 0.15)

(exp.1 + exp.2)

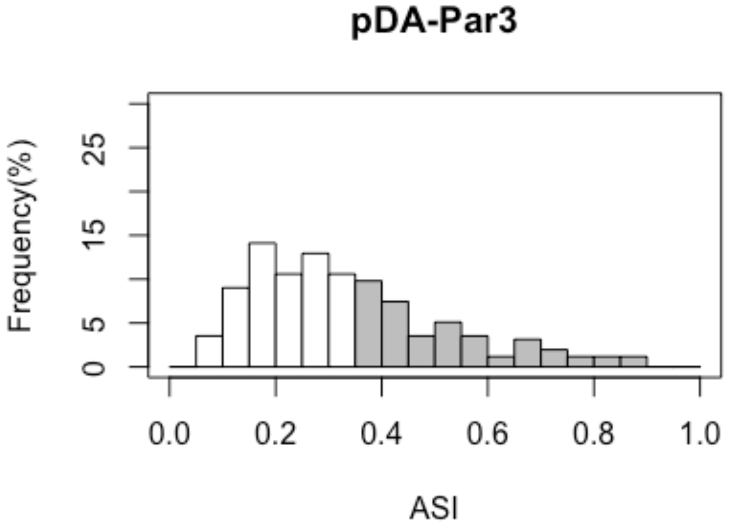


n=255
mean = 0.34, sd = 0.18
(ASI>0.35 mean = 0.52, sd = 0.14)

# UAS-Membrane

(exp.1)

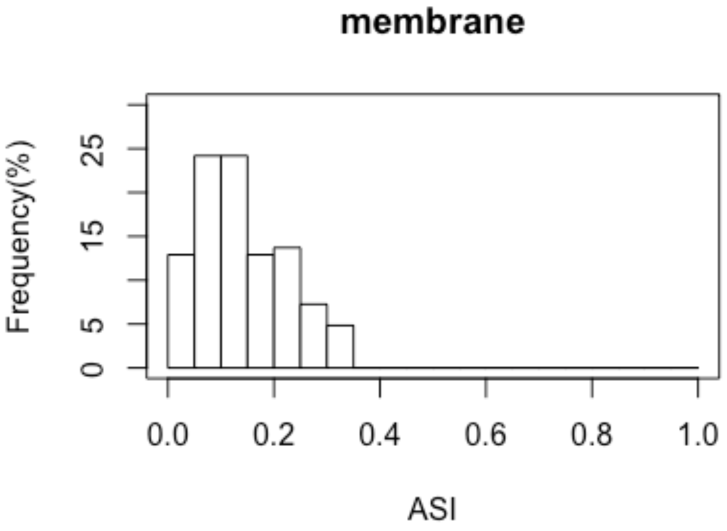


n=124
mean = 0.14, sd = 0.08

(exp.2)

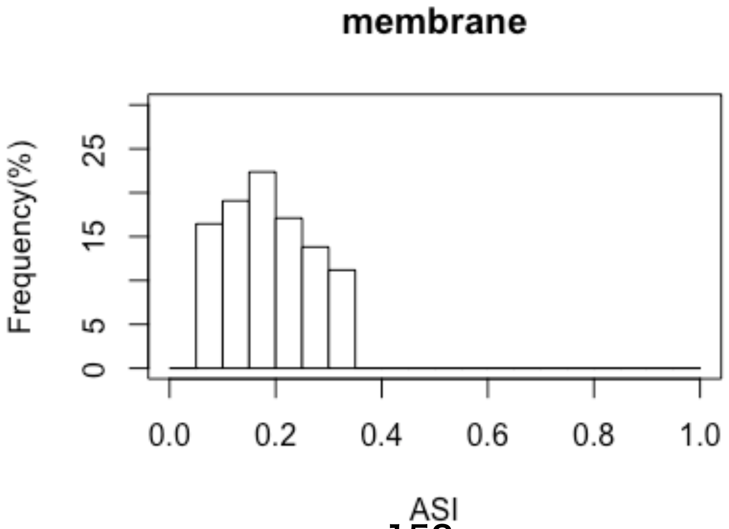


n=152
mean = 0.19, sd = 0.08

(exp.1 + exp.2)

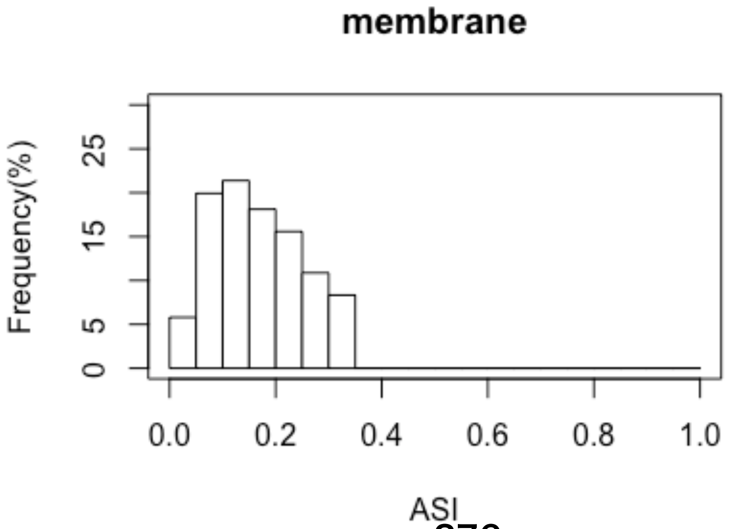


n=276
mean = 0.17, sd = 0.08

UAS-Par3 vs. UAS-membrane
ks.test < 2.2x10$^{-16}$
Wilcox.test < x10$^{-16}$
