# Supplementary material for: Reconstruction of Par-dependent polarity in apolar cells reveals a dynamic process of cortical polarization
Source: eLife. 2019 Jun 7;8:e45559. doi: 10.7554/eLife.45559 (PMC6555595; doi:10.7554/eLife.45559)

Figure12-figure supplement 2A

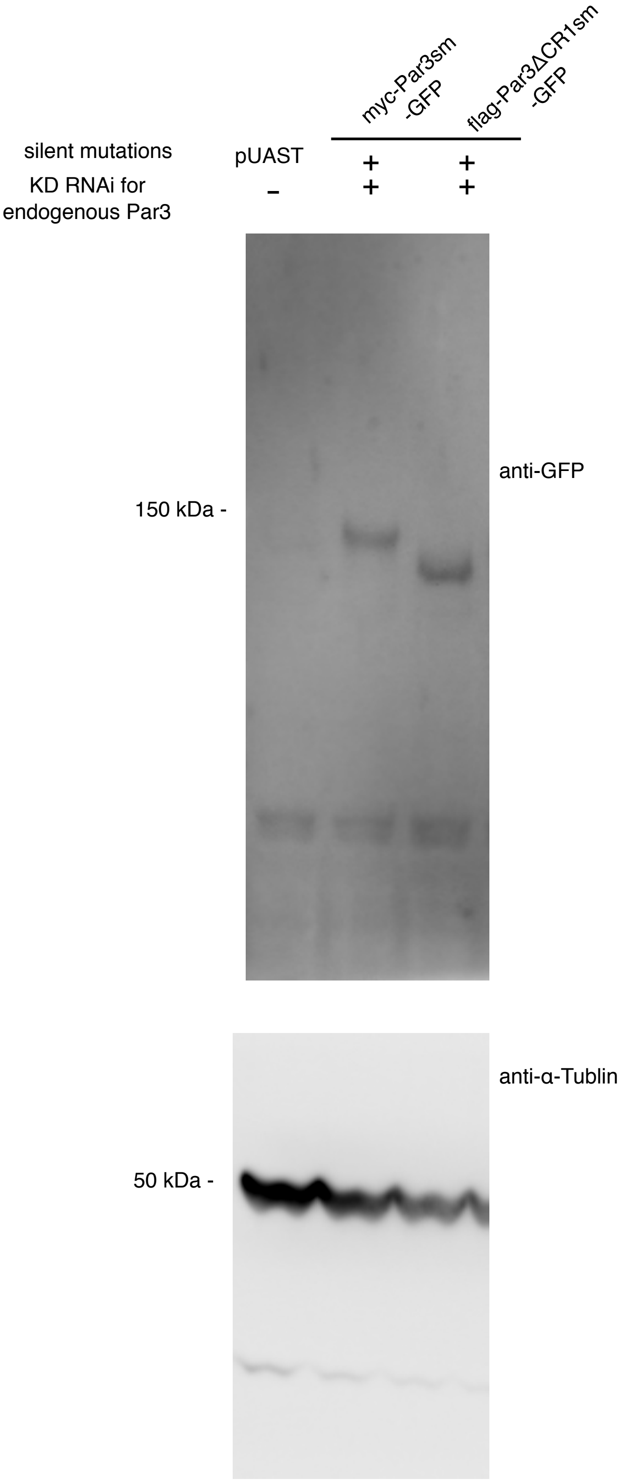

Figure12-figure supplement 2B

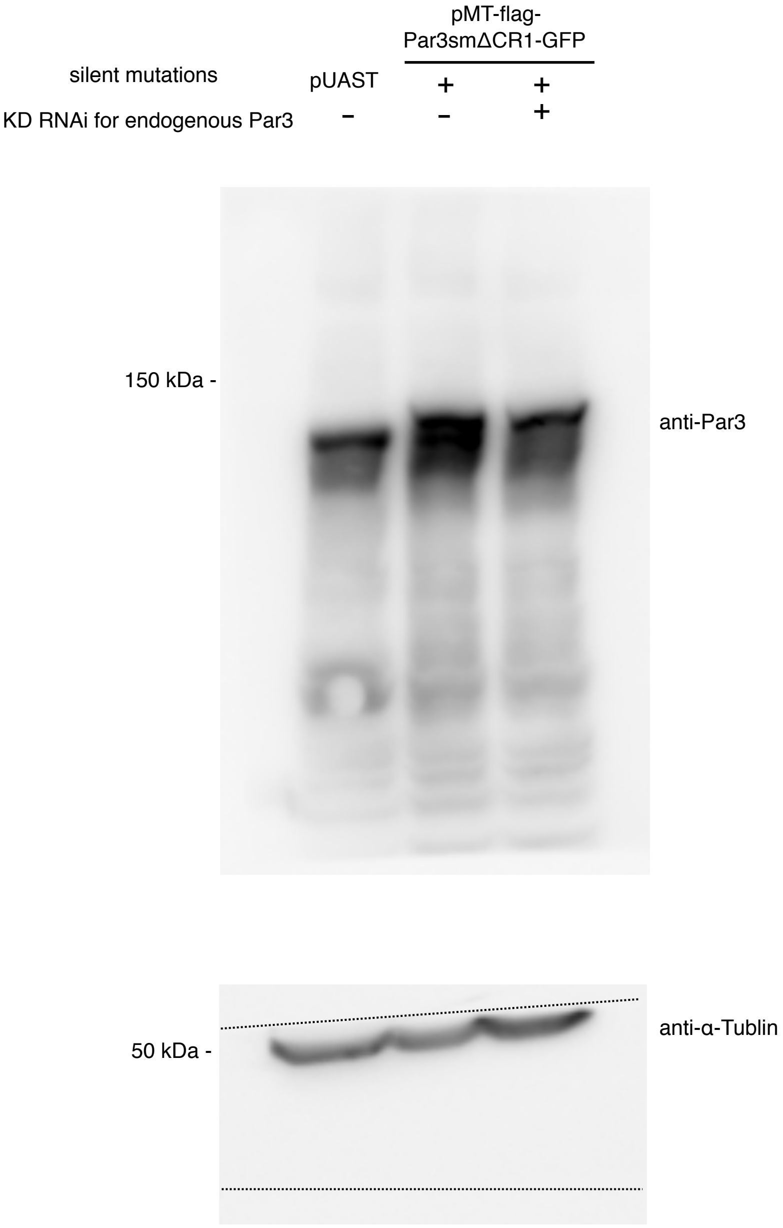

Figure12-figure supplement 2C

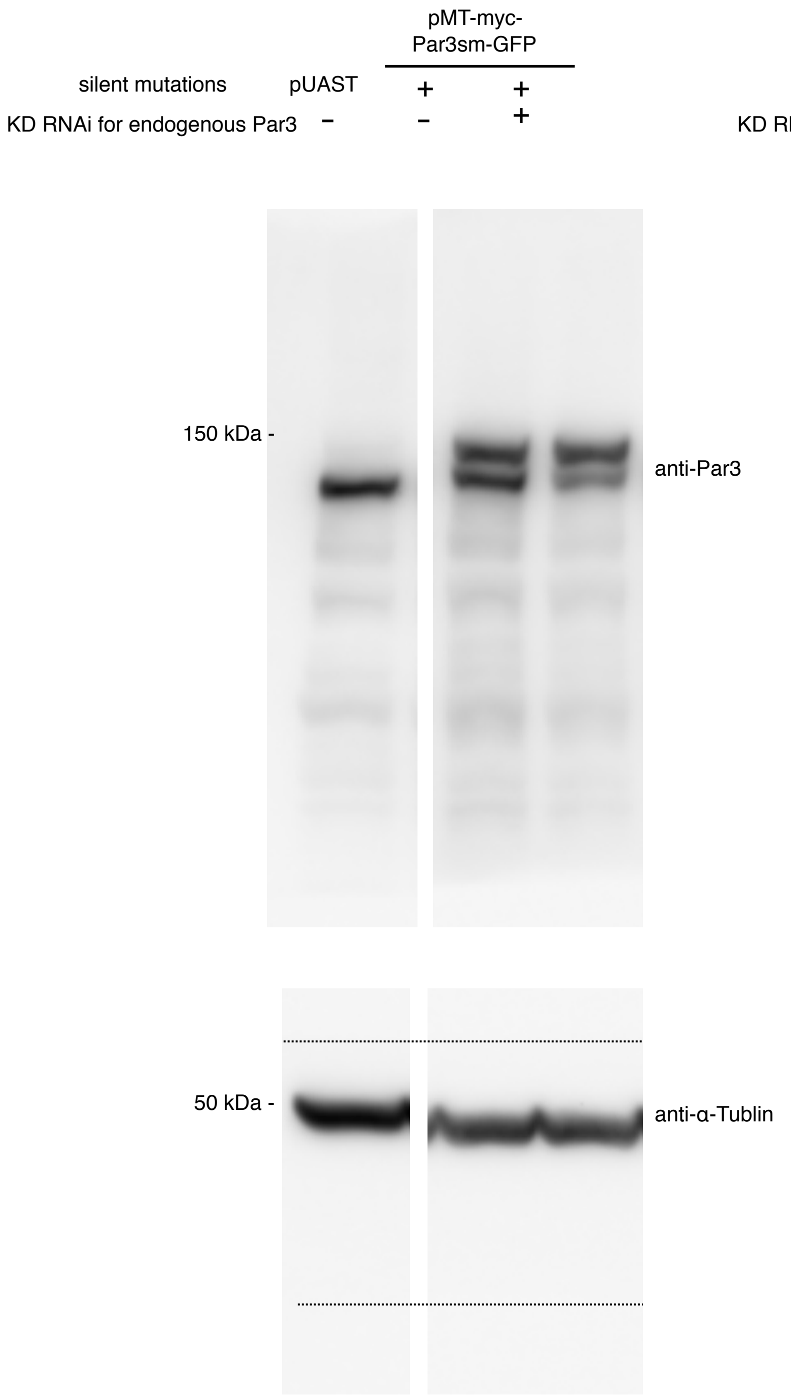

Figure12-figure supplement 2D

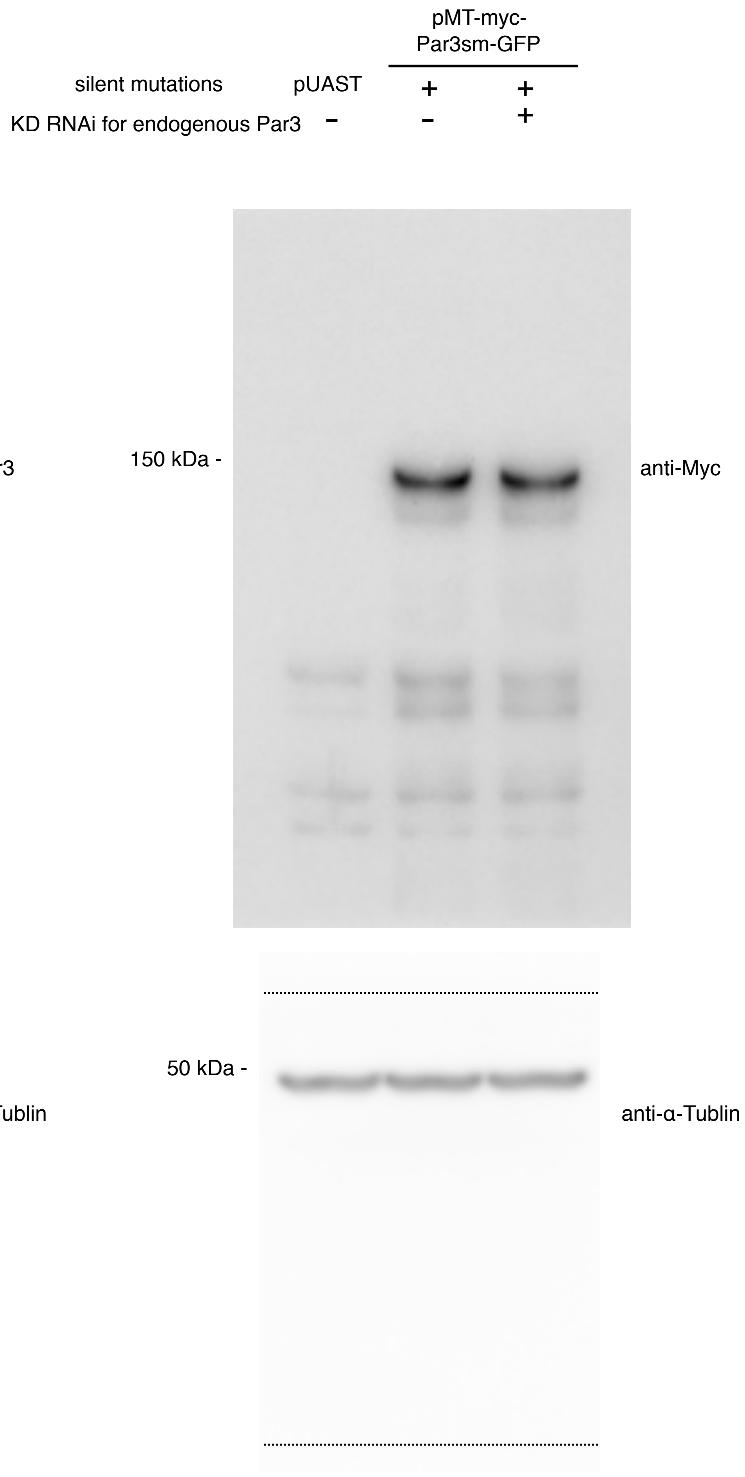

Supplement: Figure 12—figure supplement 2—source data 1. [file elife-45559-fig12-figsupp2-data1.pdf]
