# Supplementary material for: Reconstruction of Par-dependent polarity in apolar cells reveals a dynamic process of cortical polarization
Source: eLife. 2019 Jun 7;8:e45559. doi: 10.7554/eLife.45559 (PMC6555595; doi:10.7554/eLife.45559)
Supplement: Transparent reporting form [file elife-45559-transrepform.docx]

***eLife’s* transparent reporting form**

We encourage authors to provide detailed information *within their submission* to facilitate the interpretation and replication of experiments. Authors can upload supporting documentation to indicate the use of appropriate reporting guidelines for health-related research (see [EQUATOR Network](http://www.equator-network.org/%20)), life science research (see the [BioSharing Information Resource](https://biosharing.org/)), or the [ARRIVE guidelines](http://www.plosbiology.org/article/info:doi/10.1371/journal.pbio.1000412) for reporting work involving animal research. Where applicable, authors should refer to any relevant reporting standards documents in this form.

If you have any questions, please consult our Journal Policies and/or contact us: [editorial@elifesciences.org](mailto:editorial@elifesciences.org).

**Sample-size estimation**

- You should state whether an appropriate sample size was computed when the study was being designed
- You should state the statistical method of sample size computation and any required assumptions
- If no explicit power analysis was used, you should describe how you decided what s

Please outline where this information can be found within the submission (e.g., sections or figure legends), or explain why this information doesn’t apply to your submission:

- The sample size of all experiments was determined by pilot experiments, because we could not expect the results in the beginning of our study.
- We did not use statistical method to determine sample size.

**Replicates**

- You should report how often each experiment was performed
- You should include a definition of biological versus technical replication
- The data obtained should be provided and sufficient information should be provided to indicate the number of independent biological and/or technical replicates
- If you encountered any outliers, you should describe how these were handled
- Criteria for exclusion/inclusion of data should be clearly stated
- High-throughput sequence data should be uploaded before submission, with a private link for reviewers provided (these are available from both GEO and ArrayExpress)

Please outline where this information can be found within the submission (e.g., sections or figure legends), or explain why this information doesn’t apply to your submission:

1. All transfection experiments including RNAi experiments were independently duplicated, and in data analyses, the data obtained from the two experiments were combined to increase the number of samples.
2. Histograms and scatter plots. Two experiments were combined (Figure 3B, Figure7A, Figure 9B (scatter plot), Figure 12E-H and Figure 13D). The statistical values for each experiment (mean, standard deviation (s.d.), N (the number of the measurements)) are shown below.

**Figure 3B**:

pAct-Gal4>UAS-memGFP: exp1(0.14, 0.08, 124), exp2(0.19, 0.08, 151)

pAct-Gal4>UAS-Par3: exp1(0.33, 0.18, 148), exp2(0.33, 0.19, 107)

**Figure 7A**:

pAct-memGFP: exp1(0.16, 0.07, 119), exp2(0.17, 0.07, 92)

pMT-Par3: exp1(0.27, 0.14, 96), exp2(0.28, 0.14, 86)

**Figure 9B**: scatter plot & box plot of curvature radii: medians and

interquartile ranges are shown in box plots (See **Statistical reporting**).

Non-island cells: exp1(5.72, 4.57, 41), exp2(6.24, 3.99, 35)

Non-neighboring region: exp1(10.07, 27.71, 45), exp2(68.2, 365.6, 42)

Par-islands: exp1(1.79, 0.97, 39), exp2(2.21, 0.90, 30)

**Figure 12C**: exp1(0.19, 0.10, 83), exp2 (0.19, 0.11, 76)

**Figure 12F**: exp1(0.49, 0.56, 119), exp2 (1.10 ,1.03,110)

**Figure 12G** exp1(0.43,0.32,112), exp2 (0.47,0.41, 94)

**Figure 12H**: exp1(0.54, 0.54, 137), exp2 (0.50, 0.45, 68)

**Figure 13D**: exp1(0.37, 0.20, 53), exp2 (0.31, 0.17, 83).

3. The relationship of centrosome position and Par-crescent (Figure 8D). In the two independent transfection experiments, the position of centrosome was measured in the fixed section in the first experiment, while in the second experiment, centrosome position and Par crescent were measured in live imaging data, as described in the Figure 7 legend. See **Statistical reporting.**
4. Super resolution structural analyses: confocal image acquisition for SRRF processing following cell-transfection was independently duplicated (Figure 10C and Figure10-Source Data1). The histogram showing the distribution of continuous contours in the Par-complex meshwork was made by combining these two sets of data. The image acquisition by STED microscope and following analysis was once performed due to the availability of microscope.

**Statistical reporting**

- Statistical analysis methods should be described and justified
- Raw data should be presented in figures whenever informative to do so (typically when N per group is less than 10)
- For each experiment, you should identify the statistical tests used, exact values of N, definitions of center, methods of multiple test correction, and dispersion and precision measures (e.g., mean, median, SD, SEM, confidence intervals; and, for the major substantive results, a measure of effect size (e.g., Pearson's r, Cohen's d)
- Report exact p-values wherever possible alongside the summary statistics and 95% confidence intervals. These should be reported for all key questions and not only when the p-value is less than 0.05.

Please outline where this information can be found within the submission (e.g., sections or figure legends), or explain why this information doesn’t apply to your submission:

All numerical data were shown with the mean value and standard deviation (s.d.) in the form of the mean value ± s.d. when this set appears in the text and figure legends. Figure 9B is only one exception, which shows the median and number of measurements in the legend. In box plots used in Figure 9B and 11G, a box, a thick bar, whiskers and dots indicate interquartile range, median, the most extreme data points no more than 1.5 times the interquartile range from the box and data points outside the whiskers’ range, respectively.

In the initial appearance of the form of ‘the mean value ± s.d.’ in the figures (Figure 3B) and the text (page 8, line 21), we stated that in the following cases of these, the numerical value after “± “ is always s.d.

Statistical tests were done in the following data.

**Figure 6A**, retrospective comparison of the temporal change in the asymmetric index (ASI) between the cells with ASI~0.4 (asymmetric) and those cells with ASI~0.2 (non-asymmetric) at 8hr after induction of Par3. Because we compared the average ASI value of the individual 13 cells of the former type and that of individual 10 cells of the latter type, we applied t-test for statistical comparison with non-equal variances, assuming that the ASI value in the mother group of sampling cells follows the normal distribution.

**Figure 12C and13D**. we compared the ASI distribution between Par3 transfectants (Figure 6) and Par3ΔCR1-transfectants and Par3 S980A mutant transfectants. To test whether the percentage of cells with ASI<3.5 and those with ASI>3.5 are significantly different, we used Fisher’s exact test with post-hoc Bonferoni correction for comparison.

**Figure 9B, Figure 12F-G**. We compared the distributions of numerous values obtained under two different experimental conditions. Because the mother groups of these sample sets cannot be assumed to follow the normal distribution, we applied non-parametric Kolmogorov-Smirnov test for comparisons.

**Figure 8D.** We have also compared data from fixed section and live imaging regarding the relationship between centrosome position and Par crescent.

Experiment 1 (fixed sections): centrosome position within Par crescent fan: 20 cells, vs. out of Par crescent fan: 5 cells.

Experiment 2 (live imaging): centrosome position within Par crescent fan: 12 cells, vs, out of Par crescent fan: 3 cells.

Comparison with those two sets of data were done using Fisher’s exact test and results in “no significant difference with the P value of 1.0. This analysis indicates two different ways to measure the position of the centrosome relative to the Par crescent fan gave the same conclusion that the position of the centrosome is significantly correlated with the Par crescent fan.

Because this statistical analysis was not included in the manuscript, we mentioned to it here.

All raw data except Figure 1 and 2 were submitted as source data set. There was no experiment with N per group below 10.

(For large datasets, or papers with a very large number of statistical tests, you may upload a single table file with tests, Ns, etc., with reference to sections in the manuscript.)

**Group allocation**

- Indicate how samples were allocated into experimental groups (in the case of clinical studies, please specify allocation to treatment method); if randomization was used, please also state if restricted randomization was applied
- Indicate if masking was used during group allocation, data collection and/or data analysis

Please outline where this information can be found within the submission (e.g., sections or figure legends), or explain why this information doesn’t apply to your submission:

Our study is nothing to do with this issue because our study deals with *Drosophila* cultured cell, Schneider cells.

**Additional data files (“source data”)**

- We encourage you to upload relevant additional data files, such as numerical data that are represented as a graph in a figure, or as a summary table
- Where provided, these should be in the most useful format, and they can be uploaded as “Source data” files linked to a main figure or table
- Include model definition files including the full list of parameters used
- Include code used for data analysis (e.g., R, MatLab)
- Avoid stating that data files are “available upon request”

Please indicate the figures or tables for which source data files have been provided:

- We submitted all numerical data as Source data except for those included in Figure 1 and 2.s
- We did not make model construction in this study.
